# Preliminary study on the efficacy of rituximab in the treatment of idiopathic membranous nephropathy: A single-centre experience

**DOI:** 10.3389/fendo.2023.1044782

**Published:** 2023-02-15

**Authors:** Ping Chen, Min Mao, Chendan Wang, Xu Zhang, Xiaoyu Zhao, Yuanyuan Gao, Yankun Luo, Yun Zhou

**Affiliations:** ^1^ Department of Nephrology, Fifth Hospital of Shanxi Medical University (Shanxi Provincial People’s Hospital), Taiyuan, China; ^2^ Shanxi Genetic Engineering Center for Experimental Animal Models, Taiyuan, China; ^3^ Department of Nephrology, Shanxi Province Integrated Traditional and Western Medicine Hospital, Taiyuan, China

**Keywords:** idiopathic membranous nephropathy, initial treatment, refractory/relapse, rituximab, efficacy

## Abstract

**Objective:**

To investigate the efficacy of rituximab in the treatment of idiopathic membranous nephropathy (IMN).

**Methods:**

A total of 77 patients with IMN diagnosed in both our hospital and other hospitals were included in this study; the patients were divided into two groups: a treatment-naïve group (*n* = 19) and a refractory/relapsed group (*n* = 58). The clinical data of the patients, including urine examination, blood test, safety evaluation and efficacy evaluation results, were analysed retrospectively. The changes in clinical biochemical indexes and adverse reactions were compared between the two groups before and after treatment, and the clinical efficacy of rituximab (RTX) in the treatment of primary IMN and refractory recurrent membranous nephropathy was evaluated.

**Results:**

Of the 77 patients included in this study, the average age was 48 years, and there was a male-to-female ratio of 61:16. There were 19 cases in the initial treatment group and 58 cases in the refractory/relapse group. The 24-hour urine protein quantification, cholesterol, B cell count and M-type phospholipase A2 receptor (PLA2R) results in the 77 patients with IMN after treatment were all lower than those before treatment, and the differences were statistically significant (*P* < 0.05). Serum albumin was higher than before treatment, and the difference was statistically significant (*P* < 0.05). The total remission rate in the initial and refractory/relapsed treatment groups was 84.21% and 82.76%, respectively. There was no statistical difference in the total remission rate between the two groups (*P* > 0.05). During treatment, nine patients (11.69%) experienced infusion-related adverse reactions, which were relieved rapidly after symptomatic treatment. The anti-PLA2R antibody titre of the refractory/relapsed group was significantly negatively correlated with serum creatinine (*r* = −0.187, *P* = 0.045) and significantly correlated with 24-hour urine protein (*r* = −0.490, *P* < 0.001). There was a positive correlation and a significant negative correlation with serum albumin (*r* = −0.558, *P* < 0.001).

**Conclusions:**

Regardless of whether RTX is used as an initial therapy or refractory/relapsed membranous nephropathy, most patients with IMN have complete or partial remission after RTX treatment, with mild adverse reactions.

## 1 Introduction

Nephrotic syndrome caused by idiopathic membranous nephropathy (IMN) is one of the main causes of end-stage renal disease (1, 2). The clinical features of IMN are complex and varied, and the prognosis is variable. About 20% to 25% of patients with IMN can spontaneously enter remission, while about 40% of patients develop end-stage renal disease after 10 years (3).

In recent years, with the discovery of the anti-PLA2R antibody (PLA2R-Ab) and the anti-thrombospondin 7A domain antibody (THSD7A-Ab), IMN has been regarded as an organ-specific autoimmune disease (4). Autoantibodies to M-type PLA2R are specific markers of IMN, and preliminary data suggest that the anti-PLA2R antibody titre correlates with the disease’s activity (5).

Its main pathogenesis is that T cells secrete a variety of cytokines, such as interleukin, to stimulate the proliferation and activation of B cells; these B cells mediate the secretion of antibodies that bind to the podocyte surface antigens PLA2R and THSD7A to form immune complexes deposited under glomerular epithelial cells, thereby damaging the filter. Over the barrier, causing proteinuria (6). Studies have shown that glucocorticoid and cyclophosphamide regimens are effective in 60% to 70% of patients, but they have clinically significant toxic effects (7). Although calcineurin inhibitors (cyclosporine and tacrolimus) have been shown to induce the remission of nephrotic syndrome in about 70% of patients, the main limitation of these agents is the high rate of relapse after discontinuation (8). As a monoclonal antibody to CD20 on the surface of B cells, rituximab (RTX) can effectively remove B cells, block the production of antibodies and interfere with the pathogenesis of IMN (9, 10). Low-dose RTX can effectively reduce the number of B cells and the level of the PLA2R antibody in patients with membranous nephropathy (11, 12).

This breakthrough discovery provides a strong theoretical basis for using RTX in the treatment of IMN. However, in clinical trials, only 30% of patients had a history of immunosuppressive therapy, which may not represent the use of RTX in patients with other immunosuppressive therapy failures (13). To evaluate the efficacy and safety of RTX treatment, the subjects in the present study included newly treated and refractory/relapsed patients with IMN.

## 2 Materials and methods

### 2.1 Research subjects

From 1 July 2019 to 31 March 2022, 77 patients were selected as research subjects. Some of these individuals were first diagnosed with IMN by renal pathological biopsy in our hospital, and others were diagnosed with IMN in other hospitals. Renal tissue pathology is the gold standard for the diagnosis of IMN.

Inclusion criteria: (1) All selected subjects met the diagnostic criteria for nephrotic syndrome, excluding secondary factors, and were diagnosed with IMN. (2) Age = 18–80 years. (3) Baseline PLA2R antibody positive. (4) The effects of RTX drugs and the related risks were explained to patients and their families, and signed informed consent was obtained. (5) Routine urine, biochemistry, 24-hour urine protein quantification, CD20+ cell count, PLA2R antibody and other indicators were obtained before the administration of drugs.

Exclusion criteria: (1) Secondary membranous nephropathy from autoimmune diseases (systemic lupus erythematosus, Sjögren’s syndrome), chronic viral hepatitis (such as hepatitis B), endocrine and metabolic diseases (such as diabetes), malignant tumours (such as multiple myeloma), etc. (2) Drugs (such as non-steroidal anti-inflammatory drugs or heavy metals). (3) A clear diagnosis of other nephrotic syndromes.

The 77 study patients were divided into two groups according to whether RTX was the initial treatment. Those who initially received RTX were assigned to a treatment-naïve group (*n* = 19), and those who had previously received ineffective immunotherapy or had relapsed after remission were assigned to a refractory/relapsed group (*n* = 58). This study was approved by the ethics committee, and the enrolled patients gave informed consent to participate.

### 2.2 Research methods

All patients included in this study recorded their previous treatment regimens (types of glucocorticoids/immunosuppressants and medication regimens) and any infectious complications that had occurred within six months before admission. Their clinical data were collected. After admission, their condition was reassessed. After the infection was treated, RTX treatment was provided in a treatment plan. For induction therapy, RTX was administered as a single intravenous dose of 375 mg/m^2^ body surface area weekly for four weeks. For maintenance therapy, RTX was administered if proteinuria was reduced by ≥ 25% from baseline without complete remission at six months and the CD19+ B cell count was > 5. If complete remission was observed at six months, a second course of treatment was not required. Rituximab was discontinued in patients and considered a treatment failure if proteinuria decreased by < 25% within six months. All patients were followed up for more than six months.

### 2.3 Observation indicators

There were a number of factors that were monitored during the tracking period. (1) Urine examinations employed a urine routine that included 24-hour urine protein quantification. (2) Blood tests studied blood creatinine, blood urea nitrogen, blood uric acid, plasma albumin, cholesterol, triglyceride, PLA2R-Ab, B lymphocyte count, etc. (3) To provide a safety evaluation, eGFR was calculated using the CKD-EPI equation (14). Adverse events during follow-up were recorded. Follow-up endpoints were death, maintenance haemodialysis and end-stage renal disease (eGFR < 15 mL/min for more than three months). (4) Efficacy evaluations were also considered as follows: (a) Complete remission: Upro < 0.3 g/d, ALB > 30 g/l, normal renal function. (b) Partial remission: Upro = 0.3–3.5 g/d, 50% lower than before treatment % or more, ALB ≥ 30 g/L, stable renal function. (c) Ineffective: Upro decreased by less than 50% compared with before treatment, ALB < 30 g/L, or deteriorating renal function. (d) Relapse: Complete or partial remission in patients with Upro > 3.5g/d or > 50% of the baseline value (15). These indicators were collected *via* inpatient follow-up queries, the hospital’s inpatient electronic medical record system, the outpatient system and telephone follow-ups.

### 2.4 Statistical analysis

All data were statistically processed using SPSS 25.0 (Chicago, IL, USA) software. Enumeration data were expressed as frequency or percentage, and the χ^2^ test was used for comparisons between groups. Measurement data that conformed to a normal distribution were expressed as mean ± standard deviation, and a paired *t*-test was used to evaluate intergroup differences in measurement parameters. Measurement data that did not conform to a normal distribution were expressed as a median interquartile range, and nonparametric differences between groups were assessed using a U test. The association of the anti-PLA2R antibody titre with proteinuria, serum creatinine and serum albumin was analysed using a Spearman correlation analysis. A value of *P* < 0.05 indicated that a difference was statistically significant.

## 3 Results

### 3.1 Comparison of baseline data of patients with IMN between the two groups before and after treatment

Among the 77 patients in this study, 19 were in the RTX-naïve group and 58 were in the refractory or relapsed group. There were 13 males and 6 females in the newly treated group, with an average age of 48.0 years. There were 48 males and 10 females in the refractory or relapsed group, with an average age of 63.0 years (see **Table 1**).

**Table 1:** Comparison of baseline data of two groups of patients.

	New treatment group (n=19)	Refractory/relapsed group (n=58)	X^2^/t/U	*P* value
Gender: (Male/Female)	13/6	48/10	1.787	0.181
Age (years)	48.00 ± 16.31	63.0 ± 13.18	6.383	<0.001
Creatinine (μmol/L, Mdian (IQR)	82.56 (67.72,102.65)	90.78 (71.22,126.42)	2.012	0.064
Urea nitrogen (mmol/L, Mdian (IQR))	5.29 (4.52,6.95)	6.09 (5.16,9.73)	0.915	0.768
eGFR[mL·min^-^1· (1.73 m2) ^-^1, Mean ± SD]	90.24 ± 47.93	80.32 ± 31.09	1.936	0.087
24 h urine protein quantification (g/d, Mdian (IQR))	6.3 (4.64.12.31)	8.39 (4.73,11.82)	1.621	0.073
Uric acid (μmol/L, Mean ± SD)	432.66 ± 90.89	368.80 ± 93.69	8.281	<0.001
Albumin (g/L, Mean ± SD)	23.88 ± 5.31	24.66 ± 5.82	0.673	0.612
Cholesterol (mmol/L, Mean ± SD)	6.78 ± 1.33	6.62 (5.24, 7.59)	0.884	0.781
Triglycerides (mmol/L, Mean ± SD)	2.41 ± 1.21	2.23 (1.58,2.93)	0.911	0.753
B cell count (pcs/ul, Mean ± SD)	252.52 ± 123.76	146.42 (54.6, 316.35)	11.377	<0.001

The median follow-up time of the patients was 10 months (mean: 13.27 months). There were significant differences in serum albumin, cholesterol, triglycerides, B cell count and PLA2R (*P* > 0.05), but there were no significant differences in other aspects (*P* > 0.05) (see **Table 2**).

**Table 2:** Comparison of clinical data of patients with IMN before and after treatment.

Clinical indicators	Before therapy (n=77)	After treatment (n=77)	*P* value
**Creatinine (μmol/L, Mdian (IQR))**	88.55 (70.63, 126.21)	80.70 (66.20, 108.30)	0.328
**Urea nitrogen (mmol/L, Mdian (IQR))**	5.86 (4.81,9.17)	6.18 (4.79,6.18)	0.799
**eGFR[mL·min^-^1· (1.73 m2) ^-^1, Mdian (IQR)]**	83.59 (55.24,105.39)	85.545 (64.632,111.28)	0.369
**24h urine protein quantification (g/L,Mdian (IQR))**	7.37 (4.64,11.91)	2.50 (0.85,5.91)	0.001
**Uric acid (μmol/L,Mean ± SD)**	384.56 ± 96.48	384.34 ± 85.33	0.835
**Albumin (g/L,Mean ± SD)**	24.47 ± 5.67	33.79 ± 7.44	0.001
**Cholesterol (mmol/L, Mdian (IQR))**	6.56 (5.37,7.75)	5.25 (4.34,5.90)	0.001
**Triglycerides (mmol/L, Mdian (IQR))**	2.30 (1.65,3.26)	1.98 (1.65,2.65)	0.029
**B cell count (pcs/ul, Mdian (IQR))**	196.08 (73.68,304.52)	2.250 (0,12.43)	0.001
**PLA2R-Ab (RU/ml, Mdian (IQR))**	52.64 (25.72,146.365)	0 (0,11.40)	0.001

### 3.2 Comparison of the two groups before and after treatment

There were significant differences in 24-hour urine protein quantification, serum albumin, cholesterol, B cell count and PLA2R in the initial treatment group before and after treatment (*P* < 0.05). The differences in protein, triglyceride, B cell count and PLA2R were statistically significant (*P* < 0.05) (see **Table 3**).

**Table 3:** Comparison of clinical data before and after treatment between the two groups of patients.

Clinical indicators	Initial treatment group			Refractory/relapsed group		
	Before therapy (n=19)	After treatment (n=19)	*P value*	Before therapy (n=58)	After treatment (n=58)	*P value*
**Creatinine (μmol/L, Mdian (IQR))**	82.56 (67.72,102.65)	75.02 (65.75,92.95)	0.563	90.78 (71.22,126.42)	84.39 (67.00,116.20)	0.456
**Urea nitrogen (mmol/L, Mdian (IQR))**	5.29 (4.52,6.95)	5.48 (4.34,7.25)	0.773	6.09 (5.16,9.73)	6.31 (4.92,8.34)	0.685
**eGFR[mL·min^-^1· (1.73 m2) ^-^1, Mdian (IQR)]**	90.24 ± 47.93	94.66 ± 40.58	0.761	80.32 ± 31.09	85.39 ± 37.17	0.428
**24h urine protein quantification (g/L,Mdian (IQR))**	6.3 (4.64.12.31)	1.84 (0.85,3.24)	0.001	8.39 (4.73,11.82)	2.59 (0.91,6.57)	0.001
**Uric acid (μmol/L,Mean ± SD)**	432.66 ± 90.89	397.76 ± 83.49	0.226	368.80 ± 93.69	375.96 ± 98.98	0.690
**Albumin (g/L,Mean ± SD)**	23.88 ± 5.31	35.27 ± 6.74	0.001	24.66 ± 5.82	33.31 ± 7.65	0.001
**Cholesterol (mmol/L, Mdian (IQR))**	6.78 ± 1.33	5.24 ± 0.99	0.001	6.62 (5.24, 7.59)	5.20 (4.28,6.03)	0.089
**Triglycerides (mmol/L, Mdian (IQR))**	2.41 ± 1.21	2.03 ± 0.72	0.234	2.23 (1.58,2.93)	1.99 (1.64,2.34)	0.001
**B cell count (pcs/ul, Mdian (IQR))**	252.52 ± 123.76	28.68 ± 61.24	0.001	146.42 (54.6, 316.35)	2.34 (0, 9.18)	0.001
**PLA2R-Ab (RU/ml, Mdian (IQR))**	61.71 (32.23, 157.49)	0 (0, 2.57)	0.001	48.83 (27.76, 133.43)	0 (0, 25.95)	0.001

### 3.3 Comparison of the overall remission rate between the two groups

In the comparison of the remission rate between the initial treatment group and the refractory/relapsed group during the follow-up period, 14 cases achieved complete remission and 50 cases achieved partial remission, with a total remission rate of 83.12%. In the initial treatment group, two cases had complete remission, 14 cases had partial remission, and the total remission rate was 84.21%. In the refractory/relapsed group, 12 cases had complete remission, 36 cases had partial remission, and the total remission rate was 82.76%. There was no statistical difference in the total remission rate between the two groups (*P* > 0.05) (see **Table 4**).

**Table 4:** Comparison of treatment effects between the two groups.

Relieve	Initial treatment group (n=19)	Refractory/relapsed group (n=58)	*χ* ^2^	*P*
**Complete relief n(%)**	2 (10.53)	12 (20.69)	0.994	0.319
**Partial relief n(%)**	14 (73.68)	36 (62.07)	0.848	0.357
**Total relief n(%)**	16 (84.21)	48 (82.76)	0.021	0.883

### 3.4 Adverse reactions

During the infusion process, nine patients (11.69%) developed infusion-related adverse reactions, including rash, runny nose, sneezing and dysphonia, which were quickly relieved after symptomatic treatment; therefore, RTX treatment was continued.

### 3.5 Correlation analysis of PLA2R antibody titres

**Figure 1:**
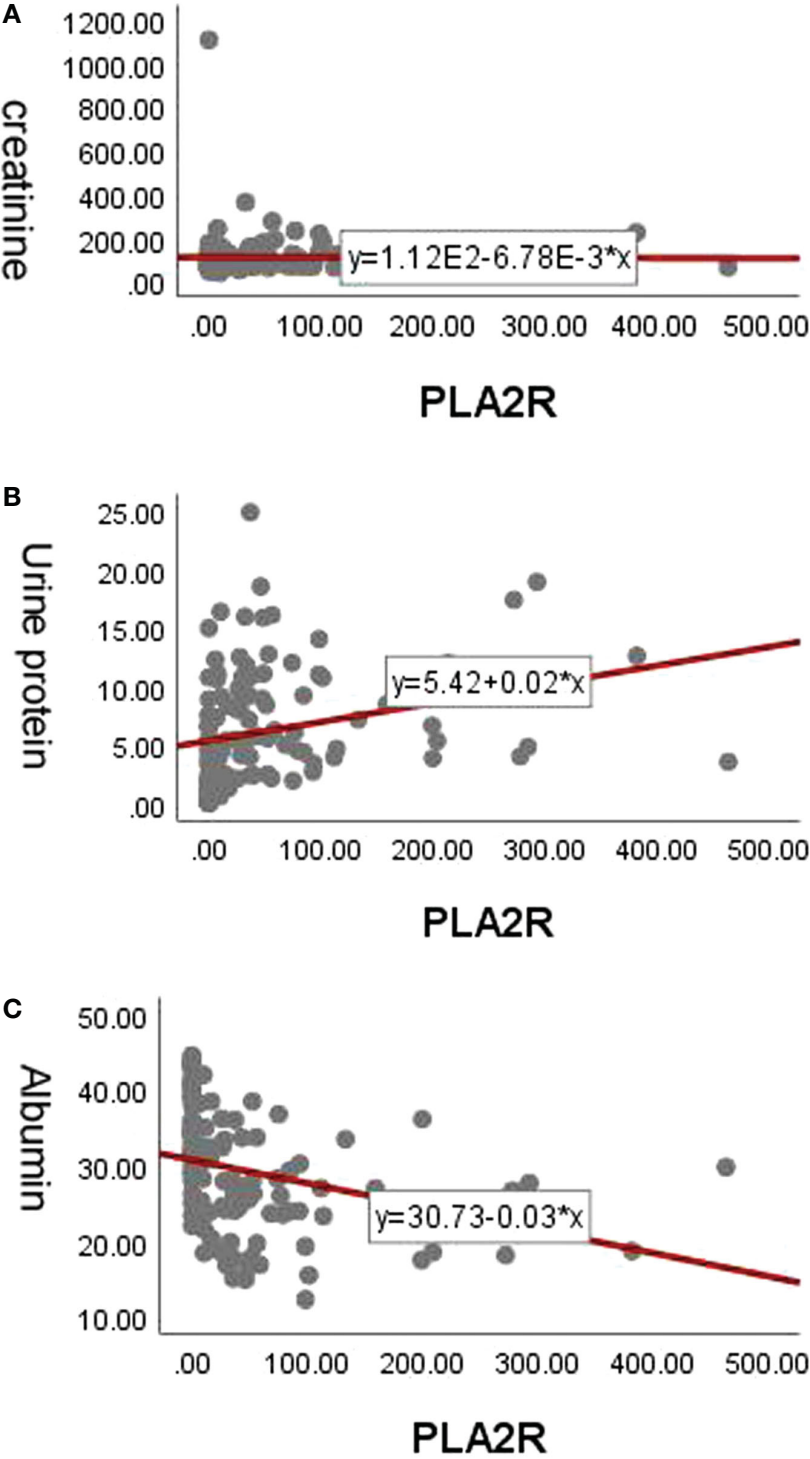
Correlation analysis of PLA2R antibody titers. **(A)** The correlation between anti-PLA2R antibody titers and serum creatinine in the refrectory/relapsed group. **(B)** The correlation between anti-PLA2R antibody titers and 24-hour urine protein in the refrectory/relapsed group. **(C)** The correlation between anti-PLA2R antibody titers and serum albumin in the refrectory/relapsed group.

The anti-PLA2R antibody titres at baseline in the treatment-naïve patients were not associated with proteinuria, serum creatinine or serum albumin (*P* > 0.05). The anti-PLA2R antibody titre in the refractory/relapsed group was significantly negatively correlated with serum creatinine (*r* = −0.187, *P* = 0.045) and significantly correlated with 24-hour urine protein (*r* = −0.490, *P* < 0.001). There was a significant negative correlation with serum albumin (*r* = −0.558, *P* < 0.001) (see **Figure 1**).

## 4 Discussion

In the past 10 years, research on the pathogenesis of membranous nephropathy has made great progress, with the detection of autoantibodies against PLA2R on glomerular podocytes in most patients with membranous nephropathy. Primary membranous nephropathy is considered to be an autoimmune disease targeting podocytes. Its possible mechanism is that the antibody binds to the podocyte surface antigen PLA2R to form an immune complex deposit under the glomerular epithelium. This compromises the filtration barrier, leading to proteinuria. The pathogenic role of autoantibody-producing B cells in membranous nephropathy has been recognised gradually, providing strong evidence for RTX in the treatment of membranous nephropathy. At present, the KDIGO guidelines regard RTX as the first-line drug for the treatment of membranous nephropathy, and some domestic experts have published a consensus opinion on the use of RTX in the treatment of membranous nephropathy to provide some guidance.

In patients with IMN, several observational studies have reported the safety and efficacy of RTX (16–19). A randomised noninferiority clinical trial (MENTOR) published in 2018 compared the use of RTX and cyclosporine in patients with membranous nephropathy. Rituximab was non-inferior to ciclosporin in inducing the complete or partial remission of proteinuria at 12 months (60% vs 52%) and was superior in maintaining the remission of proteinuria for up to 24 months (60% vs 20%) (11). Another randomised open-label controlled clinical trial (SARMEN) concluded that treatment with the traditional Ponticelli regimen resulted in significantly improved response rates in patients compared with sequential treatment with tacrolimus and RTX (8). However, patients who had received corticosteroids or other immunosuppressants before screening and those who had not responded to prior immunosuppressants were excluded from the trial.

This is far from the reality of patients with MN treated with RTX in our clinical practice. In our study, 75.3% of patients were exposed to other immunosuppressive agents before RTX treatment; however, many current clinical trials exclude all or most patients with a history of immunosuppressive therapy, and the results may not apply to patients with severe disease. Furthermore, while cyclophosphamide-based regimens do provide rapid disease control, it is worth noting that their use is sometimes avoided or discontinued in clinical practice due to their long-term toxicity.

Nonetheless, our findings suggest that, regardless of RTX as initial therapy and in patients with refractory/relapsed membranous nephropathy after other immunosuppressants, the majority of patients with MN have complete or partial remission after RTX treatment, with an overall remission rate of 83.12%. Similar to the conclusions of previous studies (9, 12), there were significant differences in serum albumin, 24-hour urine protein quantification, B cell count and PLA2R levels before and after treatment. Compared with traditional immunosuppressants (glucocorticoid and cyclophosphamide regimens) (7), RTX had mild adverse reactions; with respect to long-term efficacy, this suggests the unique advantages of RTX in the treatment of membranous nephropathy.

In our study, anti-PLA2R antibody titres at baseline in treatment-naïve patients were not associated with proteinuria, serum creatinine or serum albumin. These results are consistent with those of Hoxha et al.; they also found no correlation between proteinuria or serum creatinine and total IgG or IgG4 anti-PLA2R antibody levels at baseline (20) and showed that PLA2R seropositivity correlates poorly with clinical phenotype. However, for the patients with refractory/relapsed MN, the anti-PLA2R antibody titre was significantly positively correlated with serum creatinine and 24-hour urine protein, and it was significantly negatively correlated with serum albumin. This suggests that patients with higher antibody levels who are taking other immunosuppressive agents may take longer to achieve spontaneous remission or may require more intensive immunosuppressive therapy to achieve complete or partial remission.

The adverse events identified in our study were similar to those in previously published observational studies (16, 18–20). Infusion reactions were the most frequently described adverse events in our research, with an incidence of 11.69%; since they were rapidly resolved after symptomatic treatment, RTX treatment could be continued. However, it is worth noting that most of our patients received other immunosuppressive agents concomitantly or before RTX treatment, which may have influenced the incidence of infection. The reasons for the analysis of infection may be related to the fact that most of the subjects were patients with RNS, hypoalbuminemia and malnutrition who had received long-term treatment with glucocorticoids and various immunosuppressive agents. Therefore, this population is at high risk of infection. In addition, the patients with refractory/relapsed MN did not have a significant increase in severe infections after RTX therapy compared with the rate in the group of treatment-naïve patients, suggesting that RTX therapy does not increase the incidence of infectious complications in this population; this result is consistent with the findings of most previous studies (3, 21–26).

This study may have the following limitations: (1) It was an observational study with a retrospective design, and as the sample size was small, the results may be biased. (2) Our research was single-centred; therefore, our results may have been subject to selection bias and cannot be extrapolated to other hospitals in other geographic regions. (3) The specific correlation between the PLA2R-Ab antibody titre and the intensity of IgG4 deposition in renal tissue and the curative effect could not be further explored. (4) The levels of PLA2R-Ab antibody and CD19 were not regularly monitored during follow-up to better evaluate the efficacy. The above shortcomings represent the direction of our team’s future research efforts.

## 5 Conclusion

In summary, most patients achieved complete or partial remission after RTX treatment regardless of whether the RTX was used as an initial treatment for IMN or refractory/relapsed membranous nephropathy. Only a very small number of patients had mild adverse reactions.

## Data availability statement

The original contributions presented in the study are included in the article/supplementary material. Further inquiries can be directed to the corresponding author.

## Ethics statement

The studies involving human participants were reviewed and approved by ethics committee of Shanxi Provincial People’s Hospital. The patients/participants provided their written informed consent to participate in this study.

## Author contributions

Conception and design of the work: PC; Data collection: MM, CW, XZ, XYZ, YG, YL, and YZ; Supervision: PC; Analysis and interpretation of the data: PC, MM, CW, XZ, XYZ, YG, YL, and YZ; Statistical analysis: PC and YG; Drafting the manuscript: PC; Critical revision of the manuscript: all authors. All authors contributed to the article and approved the submitted version.
